# Inhibition of *Haemonchus contortus* larval development by fungal lectins

**DOI:** 10.1186/s13071-015-1032-x

**Published:** 2015-08-19

**Authors:** Christian Heim, Hubertus Hertzberg, Alex Butschi, Silvia Bleuler-Martinez, Markus Aebi, Peter Deplazes, Markus Künzler, Saša Štefanić

**Affiliations:** Institute of Parasitology, University of Zurich, Winterthurerstrasse 266a, 8057 Zurich, Switzerland; Malcisbo AG, Wagistrasse 27a, 8952 Schlieren, Switzerland; Institute of Microbiology, Swiss Federal Institute of Technology (ETH) Zürich, 8093 Zürich, Switzerland

**Keywords:** *Haemonchus contortus*, Fungal lectins, Nematotoxicity, Glycan targets, Vaccine development, Larval development test (LDT)

## Abstract

**Background:**

Lectins are carbohydrate-binding proteins that are involved in fundamental intra- and extracellular biological processes. They occur ubiquitously in nature and are especially abundant in plants and fungi. It has been well established that certain higher fungi produce lectins in their fruiting bodies and/or sclerotia as a part of their natural resistance against free-living fungivorous nematodes and other pests. Despite relatively high diversity of the glycan structures in nature, many of the glycans targeted by fungal lectins are conserved among organisms of the same taxon and sometimes even among different taxa. Such conservation of glycans between free-living and parasitic nematodes is providing us with a useful tool for discovery of novel chemotherapeutic and vaccine targets. In our study, a subset of fungal lectins emanating from toxicity screens on *Caenorhabditis elegans* was tested for their potential to inhibit larval development of *Haemonchus contortus*.

**Methods:**

The effect of *Coprinopsis cinerea* lectins - CCL2, CGL2, CGL3; *Aleuria aurantia* lectin – AAL; *Marasmius oreades* agglutinin - MOA; and *Laccaria bicolor* lectin – Lb-Tec2, on cultivated *Haemonchus contortus* larval stages was investigated using a larval development test (LDT). To validate the results of the toxicity assay and determine lectin binding capacity to the nematode digestive tract, biotinylated versions of lectins were fed to pre-infective larval stages of *H. contortus* and visualized by fluorescent microscopy. Lectin histochemistry on fixed adult worms was performed to investigate the presence and localisation of lectin binding sites in the disease-relevant developmental stage.

**Results:**

Using an improved larval development test we found that four of the six tested lectins: AAL, CCL2, MOA and CGL2, exhibited a dose-dependent toxicity in LDT, as measured by the number of larvae developing to the L3 stage. In the case of AAL, CGL2 and MOA lectin, doses as low as 5 μg/ml caused >95 % inhibition of larval development while 40 μg/ml were needed to achieve the same inhibition by CCL2 lectin. MOA was the only lectin tested that caused larval death while other toxic lectins had larvistatic effect manifesting as L1 growth arrest. Using lectin histochemistry we demonstrate that of all lectins tested, only the four toxic ones displayed binding to the larvae’s gut and likewise were found to interact with glycans localized to the gastrodermal tissue of adults.

**Conclusion:**

The results of our study suggest a correlation between the presence of target glycans of lectins in the digestive tract and the lectin-mediated toxicity in *Haemonchus contortus*. We demonstrate that binding to the structurally conserved glycan structures found in *H. contortus* gastrodermal tissue by the set of fungal lectins has detrimental effect on larval development. Some of these glycan structures might represent antigens which are not exposed to the host immune system (hidden antigens) and thus have a potential for vaccine or drug development. Nematotoxic fungal lectins prove to be a useful tool to identify such targets in parasitic nematodes.

**Electronic supplementary material:**

The online version of this article (doi:10.1186/s13071-015-1032-x) contains supplementary material, which is available to authorized users.

## Background

At a global perspective, *Haemonchus contortus* (also known as the barber pole worm) is regarded as the major helminth pathogen of small ruminants causing extensive economic losses in sheep and goat production. The control of *H. contortus* mainly relies on the use of anthelmintics, but widespread resistance to commonly used drugs [1–5] urges the development of new chemotherapeutics and alternative control strategies such as vaccination. Research on vaccines against *H. contortus* identified a range of parasite molecules that conferred high levels of protection against challenge infection [6–10] (reviewed in [11, 12]), mostly induced by antigens of native origin [13, 14]. Recombinantly expressed analogues generally stimulate lower levels of protection than their native counterparts and inappropriate glycosylation is considered to be the most likely explanation [15]. In fact, some of the most promising vaccine candidates characterized to date, including larval antigen Hc-sL3, and adult antigens H11, H-gal-GP, contain glycan modifications that might be greatly contributing to protective immunity against *H. contortus*. H11 and H-gal-GP are believed to be components of the hemoglobinase complex in the *H. contortus* adult gut responsible for digestion of the ingested host hemoglobin and are regarded as hidden antigens not exposed to the host immune system [9, 16, 17].

Attempts were made to further characterize protective immune responses induced by *H. contortus* infection by analyzing sera of vaccinated animals on glycan microarrays in order to discover reactive glycan epitopes which might be involved in protection [18]. This approach led to the identification of novel glycan structures of *H. contortus* that are also known as natural targets of nematotoxic lectins expressed in fungal fruiting bodies [19]. Fungi use such lectins as part of their innate defence against predatory nematodes and other pests [20] and it has been hypothesized that the glycan epitopes of these lectins are often conserved between different species of the same taxon or even across different taxa [21]. Several target glycan structures of fungal defence lectins have been identified in the model nematode *Caenorhabditis elegans* [19, 22–25]. Binding of the lectins to these glycans as part of surface glycoproteins or glycolipids on the intestinal epithelium led to inhibition of larval development or sometimes even to larval death. Some of these glycans are likely to be conserved in parasitic nematode species like *H. contortus* where they may, based on their hidden nature and their immunogenicity (see above), represent ideal carbohydrate vaccine candidates. Indeed, glycans of *H. contortus* have been identified which are structurally identical with those in *C. elegans* [26–28] and some of them, like fucosylated N-glycan cores and Galα1-3GalNAc play a role in acquired immunity against *H. contortus* [18, 27, 29]. Thus, nematotoxic lectins with specificites to such conserved glycan structures may provide us with a tool to identify novel potential vaccine candidates in *H. contortus*.

In order to further validate this approach of carbohydrate vaccine identification, we implemented an *in vitro* larval development test to investigate anthelmintic properties of six fungal fruiting body lectins (Table 1; *Coprinopsis cinerea* lectins - CCL2, CGL2, CGL3; *Aleuria aurantia* lectin – AAL; *Marasmius oreades* agglutinin - MOA; *Laccaria bicolor* lectin – Lb-Tec2, referred to as Tectonin throughout the text), and used histochemistry on cryosectioned larval and adult stages of *H. contortus* to localize the lectin binding sites. Results of this study identified four fungal lectins with inhibitory activity on development of *H. contortus* larval stages (AAL, MOA, CCL2 and CGL2). All toxic lectins displayed specificity for glycan structures present in the digestive tract of the larvae, as well as on the resorptive surface of the adult parasite gut. The results of this study show that not only the structure, but also the location and the function of target glycans of fungal nematotoxic lectins are conserved between *C. elegans* and *H. contortus*.

**Table 1:** Overview of the fungal lectins used for biotoxicity assays in this study

Lectin	Origin	Lectin family	Molecular Weight	Carbohydrate specificity	References	GenBank accession number
CCL2	*Coprinopsis cinerea*	β-trefoil	15	GlcNAc-β1,4-(Fuc-α1,3-) GlcNAc	Schubert et al. [23]	ACD88750
					Stutz et al. [30]	
CGL2	*Coprinopsis cinerea*	Galectin	16.7	Galβ1-4Glc (lactose)	Walser et al. [31]	AAF34731
					Butschi et al. [22]	
				Galβ1-4GlcNAc		AAF34732
				Galβ1-4Fuc		
CGL3	*Coprinopsis cinerea*	Galectin-like	19	GlcNAcβ1-4GlcNAc	Walti et al. [32]	ABD64675
				GalNAcβ1-4GlcNAc		
AAL	*Aleuria aurantia*	β-propeller	33.4	Fucose	Fujihashi et al. [33]	BAA12871
					Wimmerova et al. [34]	
MOA	*Marasmius oreades*	β-trefoil (B-type)	33	Galα1,3Gal/GalNAc	Wohlschlager et al. [19]	AAL47680
					Cordara et al. [35]	
Tec2	*Laccaria bicolor*	β-propeller	23.8	2-O-Me-Fucose	Wohlschlager et al. [25]	EDR12168
				3-O-Me-Mannose		

Table adapted and modified from S. Bleuler-Martinez et al. 2011 [24]

## Methods

### Ethics statement

The animal experiments with sheep (*Ovis aries*) were carried out in accordance with Swiss legislation on animal protection (Animal Wellfare Act: TSchG 455) following ethical principles and guidelines for experiments on animals of the Swiss Academy of Sciences and using the protocols approved by the Cantonal Veterinary Office.

### Egg isolation and cultivation of *Haemonchus contortus* larval stages

Faeces from sheep mono-infected with a pure *Haemonchus contortus* isolate were used. Depending on the faecal egg count, 1 to 5 g of faeces were homogenised in 200 ml of tap water until all faecal pellets were broken up and completely dissolved. The eggs were cleaned from coarse particles by passing through a household sieve overlaid with 3 layers of cotton gauze and the flow-through was then filtered through a stack of sieves having 200 μm, 150 μm, 100 μm, 50 μm and 32 μm mesh diameter. Eggs from the 32 μm sieve were transferred into 50 ml Falcon tube and pelleted at 600 g for 2 min. One ml of the egg suspension was loaded on the top of the Percoll gradient (GE Healthcare Biosciences AB, SE-751 84 Uppsala) consisting of 2.5 ml layer of each 45 %, 40 %, 35 % and 30 % (from the bottom to the top) in a 15 ml tube (Sarstedt D-51588 Nuremberg). The gradient was then centrifuged at 1400 g for 15 min and eggs were recovered from the middle of the 35 % Percoll fraction. The eggs were subsequently washed four times with double-concentrated (50 x dilution of stock) antibiotic-antimycotic solution for 10 min each (Gibco® / LifeTechnologies). The egg suspension volume was adjusted with distilled water to obtain a final concentration of 5 eggs per μl.

The cultivation medium for hatched larvae contained yeast extract (Becton Dickinson & Co.), Earle’s salts (Sigma), and heat killed and lyophilized bacteria as a food source. One gram of yeast extract was dissolved in 90 ml 0.85 % NaCl, and then 3 ml of 10x Earle’s salt solution per 27 ml yeast extract was added, adjusted to pH 7.0, sterile filtered and stored frozen in aliquots. *Escherichia coli* OP 50 strain was cultivated overnight in 50 ml of LB medium at 37 °C. Optical density at 600 nm wavelength was measured and the cells were pelleted by centrifugation at 2500 g for 10 min. The bacterial cell pellet was subsequently resolved in the adequate volume of LB medium to obtain OD_600_ = 15. The bacterial suspension was heat treated at 65 °C in a water bath for 5 min, and aliquots of 0.5 ml were frozen in liquid nitrogen for lyophilisation. The lyophilised bacteria were stored at −20 °C and reconstituted with distilled sterile water to the original volume before use. The optimal amount of bacteria used for LDT was empirically obtained as described below.

### Media evaluation test

Before exposing *H. contortus* eggs and hatched larvae to fungal lectins, the effect of different culture conditions on larval vitality and development to L3 stage has been tested. Basic cultivation media contained 20 μl yeast extract in Earle’s balanced salt solution, prepared as described above, to which 1–6 μl of either sterile water, sheep faecal extract as described in [36], living bacteria (*E. coli* OP50 cultivated overnight at 37 °C in LB media), or heat treated and lyophilised bacteria (prepared as above) were added. To each well, 10 μl of egg solution containing approximately 50–60 eggs and 114–119 μl of distilled water was added to make a total volume of 150 μl per well. Cultivation was done in 96-well flat-bottom plates (TPP, Trasadingen, Switzerland) at 26 °C until day 7, when the percentage of larvae developed to L3 was visually determined by light microscopy.

### Lectin production, purification and biotin labelling

All lectins were produced and purified as described previously [19, 22–25, 32]. Briefly, corresponding cDNAs had been amplified and cloned in *Escherichia coli* strain DH5α and the proteins were expressed in *E. coli* strain BL21 (DE3). The lectins were purified either via carbohydrate affinity chromatography using appropriate carbohydrate ligands, or via polyhistidine tag (6x His) using immobilized metal-affinity chromatography (IMAC), subsequently desalted on a PD-10 column (Amersham Biosciences) and concentrated using an Amicon Ultra-4 centrifugal filter device (Millipore) with a molecular weight cut-off of 10 kDa. For fluorescence microscopy, purified lectins were labelled with EZ-Link sulfo-NHS-biotin kit (Pierce) according to manufacturer’s instructions, followed by desalting and concentration as for unlabelled lectins.

### Biotoxicity assay and statistical evaluation

Media composition and volumes were based on the media evaluation test (see above). Each well contained 103 μl of distilled water, 20 μl yeast extract in Earle’s balanced salt solution, 2 μl heat-treated and lyophilized bacteria as larval food source, 10 μl egg suspension with approximately 50 *H. contortus* eggs and either 15 μl of lectin solutions or sterile water as negative control. Each sample was tested in triplicate and the assay was performed three times. CGL2, AAL and MOA lectins were tested at final concentrations of 1, 5 and 10 μg/ml, CCL2 was tested at 10, 20 and 40 μg/ml, Tectonin and CGL3 were tested at concentrations of 10, 50 and 100 μg/ml. The concentrations were chosen on the base of preliminary toxicity assays. The plate was incubated at 26 °C and relative humidity of 100 % for 7 days and checked daily for hatching of the eggs and phenotypic assessment of the larval development under a light microscope. After 7 days the number of larvae developed to L3 stage was determined and expressed as percentage relative to the control which was set as 100 % development for the respective test.

The statistical significance of the toxicity assays was evaluated using a *t*-test for pairwise comparisons between each lectin test concentration and the untreated control.

### Fluorescence microscopy using biotinylated lectins

#### Sample preparation

Exposure of larvae to biotinylated lectins was performed in 24-well plates (TPP, Trasadingen, Switzerland) in a final volume of 1 ml. For this experiment all biotinylated lectins were tested at final concentration of 20 μg/ml. Eggs were allowed to hatch overnight and L1 were further incubated for 9 h without change of media allowing ingestion of biotinylated lectins. After that, the lectin-containing medium was replaced with 0.85 % NaCl solution and larvae were allowed to empty their gut contents during the following 24 h with two more media exchanges (0.85 % NaCl) at 8 h intervals. Finally, the larvae were fixed and prepared for histochemistry as described below for adult specimens, except for the lectin incubation step.

Adults of *Haemonchus contortus* were obtained from the abomasum of experimentally infected sheep. The worms were washed 3 times in PBS and then fixed in 4 % formaldehyde in PBS overnight at 4 °C. Following fixation, the samples were rinsed once with PBS and incubated in 30 % sucrose solution at 4 °C overnight (until they sank to the bottom of the tube). Subsequently, the sucrose solution was completely removed and the samples were embedded in O.C.T. medium (CellPath Ltd, UK) in plastic moulds and frozen on top of dry ice. The blocks were stored at −80 °C until cutting (Cryostat 2800 Frigocout, Cambridge Instruments GmbH). Sections of ~7 μm were air dried and further processed for fluorescence microscopy.

### Lectin histochemistry and microscopy

The sections were rehydrated in PBS and additionally fixed with 4 % formaldehyde 40 min at room temperature. The sections were then washed with PBS and blocked in 0.1 M glycine for 20 min at room temperature and further incubated in 2 % BSA/PBS at 4 °C overnight. Following blocking step, the adult worm sections were probed with biotinylated lectins in 2 % BSA/PBS at 5, 10 and 20 μg/ml for 45 min at room temperature, washed three times with 1 % BSA/PBS and incubated in 15 μg/ml Atto 655 Streptavidin (Sigma-Aldrich) in 2 % BSA/PBS for another 45 min at room temperature. Finally, the samples were washed 4 times with 1 % BSA/PBS (third wash contained 1 μg/ml DAPI, to stain nuclear DNA) embedded in mowiol or Vectashield (Vector Laboratories, Burlingame, CA, USA) and visualized using a Leica SP2 AOBS confocal laser scanning microscope (Leica Microsystems, Wetzlar, Germany). The acquisition settings (laser power, gain and offset) were determined on the control slide (probed with Atto 655 Streptavidin alone) by defining the signal threshold and then, without changing any of the acquisition parameters, the images were acquired from slides probed with biotinylated lectins.

The fluorescent channels were false-coloured using Zeiss LSM Image Browser and exported to CorelDRAW 12 software for preparation of the figures.

### Light microscopy of *H. contortus* larvae

Differential interference contrast (DIC) images were acquired on a Leica DMI 6000B epifluorescent microscope using a 10x/0.30 inverted objective. Image acquisition and processing was done with Leica Application Suite AF software and exported to CorelDRAW 12 for preparation of the figures.

## Results

### Media evaluation for larval cultivation

**Fig. 1:**
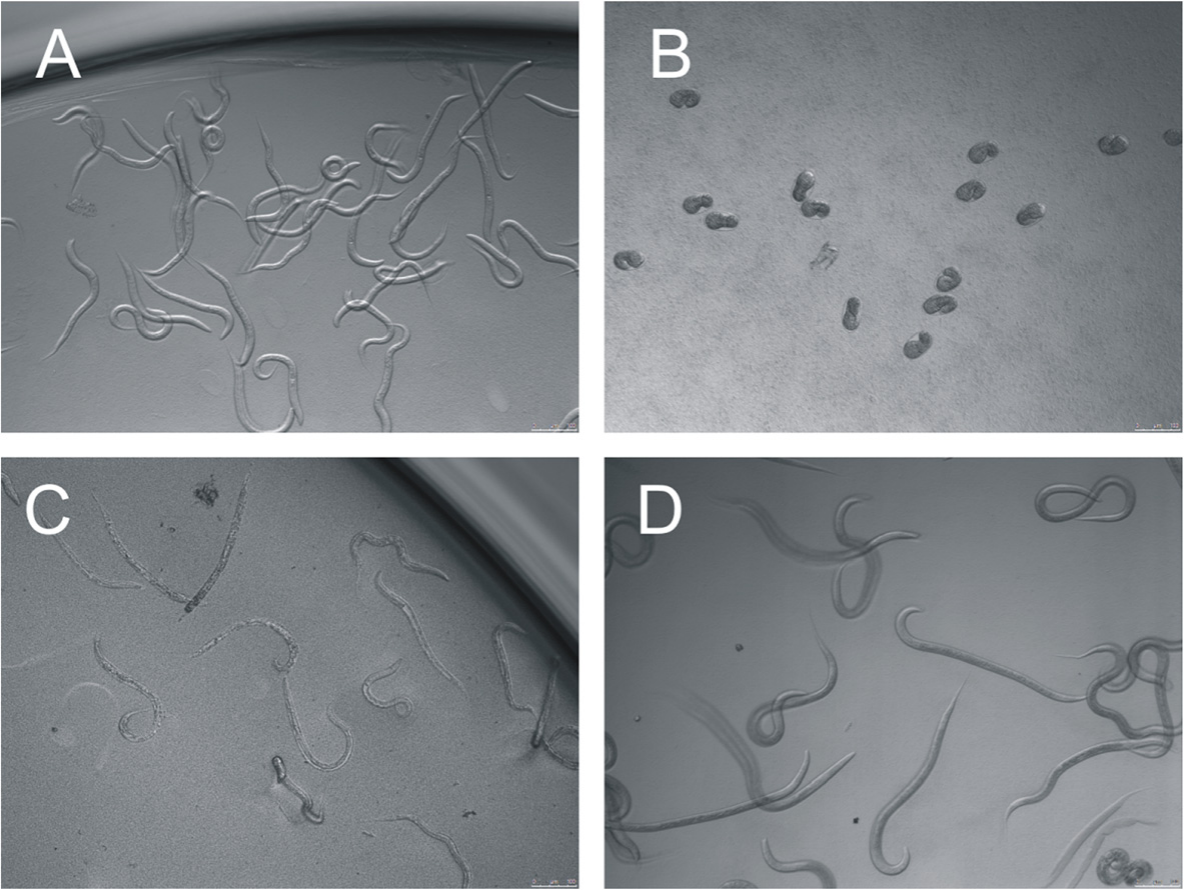
Effect of different nutritive media on *H. contortus* larval development. Basic medium containing only yeast extract and salts fails to promote larval development and the larvae stagnate in L1 stage (**a**). When sheep faecal extract from egg isolation (flow-through during sieving) is used as media supplement, none of the larvae hatch. The eggs embryonate, but the larvae die before hatching (**b**). Using living bacteria solution as a food source eventually leads to bacterial overgrowth during the cultivation period of 7 days resulting in death of the larvae and irreproducible L3 counts (**c**). Addition of heat-treated and lyophilized bacteria solution supports development of larvae to L3 stage without inducing bacterial or fungal contamination (**d**)

To prevent commonly reported problems associated with contamination of larval cultures with unknown bacteria or fungi we aimed at a cleaner egg preparation. For this purpose, the standard egg sedimentation-flotation protocol was modified to include separation of eggs over the Percoll density gradient and extensive washing of eggs in antibiotic-antimycotic solution prior to seeding in the test plates. Furthermore, different culture media compositions were compared on their suitability to promote larval development to L3 stage. Of four different media containing different sources of food: yeast extract alone (Fig. 1a), yeast extract supplemented with sheep faecal extract (Fig. 1b), and yeast extract supplemented with either living (Fig. 1c) or heat-treated and lyophilized bacterial culture of *E. coli* OP50 strain (Fig. 1d), only the latter resulted in obtaining a reproducibly high percentage of *H. contortus* larvae that developed to L3 stage within one week of *in vitro* cultivation without inducing problems associated with bacterial or fungal contamination. The improved LDT resulted in egg hatching rate of >95 % and development of larvae to L3 stages of ~75 % in average of 18 experiments performed in triplicates (range 57.57 – 88.75 %, Additional file 1: Figure S1). Based on these results we used 2.0 μl of heat-treated and lyophilized bacteria solution as food source in the lectin toxicity assay.

### Biotoxicity assay

**Fig. 2:**
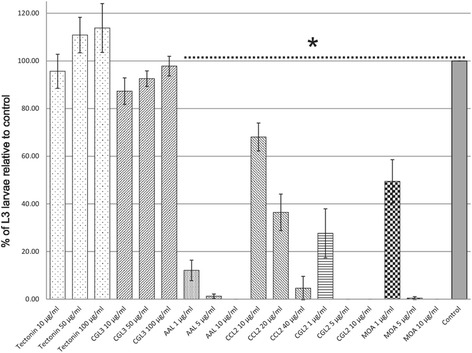
Inhibitory effect of fungal lectins on the development of *H. contortus* larval stages. The graph shows the effect of exposure of *H. contortus* L1 larvae to six fungal lectins: Tectonin, CGL3, AAL, CCL2, CGL2, and MOA. Development of *H. contortus* larvae to L3 stage was quantified relative to respective untreated control which was set as 100 %. CCL2, CGL2, AAL, and MOA inhibited development of the larvae to L3 stage in a dose-dependent manner. Bars represent the means of three independent experiments. Error bars indicate the standard deviations. Asterisk indicates statistically significant difference (*p* < 0.01)

A set of six fungal lectins was tested in a biotoxicity assay for their potential to inhibit development of *H. contortus* larval stages. Tectonin and CGL3 had no visible effect at the tested concentrations, whereas AAL, CGL2, MOA and CCL2, demonstrated an inhibitory effect on the development of *H. contortus* larvae to L3 stage (Fig. 2). The highest potency was observed in larvae exposed to CGL2, AAL and MOA, in which case lectin concentrations of only 1 μg/ml strongly inhibited larval development (CGL2 = 72 % *p* = 0.016, AAL = 88 % *p* = 0.004, and MOA = 50 % inhibition *p* = 0.014), while these three lectins in concentrations of 5 μg/ml almost completely (>98 %) prevented development to L3 stage. A dose-dependent inhibition of larval development was also observed in larvae exposed to CCL2 lectin, albeit at higher concentrations (e.g. 40 μg/ml to achieve 95 % inhibition, *p* < 0.001, Fig. 2). In the case of Tectonin and CGL3 lectins, exposure to concentrations as high as 100 μg/ml did not affect larval development during the test (*p* > 0.05). Regarding the effect of lectins on larval morphology, stunted larval growth (arrest in L1 stage) was observed in AAL, CCL2 and CGL2 lectins, without other visible alterations of phenotype or viability during one week cultivation. In contrast, the larvae exposed to MOA lectin displayed severe malformations in body shape, characterized predominantly by shrunk body appearance with undulating cuticle (Additional file 2: Figure S2). These morphological changes started being noticeable from day 4 and quickly led to death of affected larvae. The larvae exposed to Tectonin and CGL3 didn’t show any phenotypic alterations compared to the control.

### Localisation of lectin binding sites in *Haemonchus contortus*

**Fig. 3:**
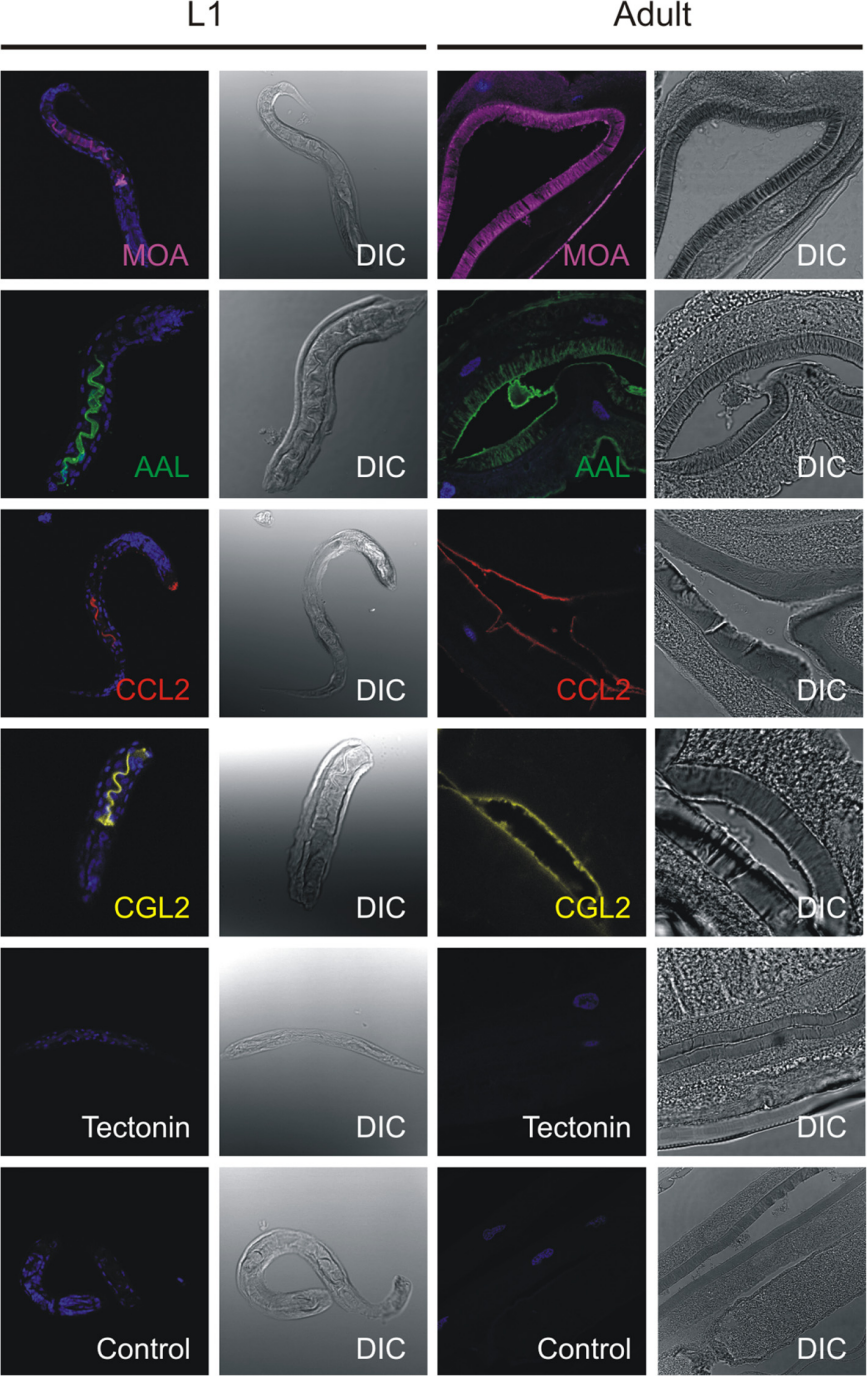
Toxic lectins bind to the gut of *H. contortus*. Indirect immunofluorescence microscopy using biotinylated lectins was performed on *in situ* labelled L1 stages and on fixed tissue sections of adults. All four toxic lectins (MOA, AAL, CCL2 and CGL2) bind to the digestive tract of larval stages distal from the oesophagus and to the brush border of adult gastrodermal cells. Additionally, CCL2 lectin also binds to the pharyngeal region of the L1 stage while MOA detects epitopes present in the inner cuticle layer of adults exposed by the sample preparation method. Tectonin does not show any fluorescent signal in the far-red channel (655λ) neither in larvae or adult stages, as well no detectable signal is present in the control larvae probed with Atto 655 Streptavidin alone. For an easier overview, the fluorescent signal from far-red channel is false-coloured differently for each lectin. Blue colour represents nuclear DNA stained with DAPI. DIC = Differential Interference Contrast

In order to determine if biotoxicity correlates with the ability of lectins to bind glycan structures present in the worm gut, indirect immunofluorescence microscopy was performed on *in situ* labelled L1 stages and on fixed sections of adults using biotinylated lectins. Larvae which were exposed to the inhibitory lectins: MOA, AAL, CCL2 and CGL2, demonstrated lectin binding to the digestive tract distal from the oesophagus (Fig. 3, leftmost column). Additionally, the CCL2 lectin bound to the pharyngeal region of the L1 stages. Larvae incubated with the non-toxic lectins, Tectonin and CGL3 or with Atto 655 Streptavidin alone (negative control), did not show any fluorescent signal (Fig. 3, CGL3 not shown).

Because of practical issues, *in situ* labelling of living *H. contortus* adults with biotynilated lectins could not be performed. In order to determine if the glycan epitopes targeted by toxic lectins in L1 larvae are also found in the gastrodermal tissue of adult worms, formaldehyde fixed semi-thin sections of adult worms were probed with biotinylated lectins. In accordance with the results of *in situ*-labelled L1 stages, all four lectins that bound the gut of L1, also labelled the brush border of the adult gut (MOA, AAL, CCL2, CGL2; Fig. 3, 3^rd^ column). However, this method of sample preparation exposed worm tissues and glycan structures which most likely would not be accessible to the lectins in living adults. For example, fasciae of internal organs in the case of AAL and one of the inner cuticle layers in the case of MOA lectin also stained positive.

In contrast to toxic lectins, probing adult tissue sections with Tectonin (Fig. 3) and CGL3 lectin (data not shown) did not result in visible staining, supporting the results obtained with *in situ*-labelled L1 stages.

## Discussion

Of the 4 tested cultivation media, addition of heat-treated and lyophilized *E. coli* was the most suitable food source for supporting *H. contortus* larval development. The basic medium containing only yeast extract and salts, originally developed for the purpose of anthelmintic screening [37], failed to promote larval development during a one-week period. The larvae hatched, but stagnated in L1 stage (Fig. 1a). Other researchers circumvented this problem by adding bacteria isolated from sheep faecal extract [36]. Despite promoting larval development, assays showed high variability depending on the amount of faecal water used due to the non-standardised composition of microflora from different sources [38]. Furthermore, toxicity assays using lectins might fail because of possible interaction of lectins with ingredients of faeces from plant origin. In our assay, addition of faecal water resulted in complete inhibition of larval hatching (Fig. 1b) which can likely be associated with extreme changes in physical conditions caused by bacterial overgrowth and accumulation of metabolic waste products. Therefore, LDT that uses faecal extract is difficult to standardise and is not regarded suitable for lectin biotoxicity assays. The same authors describe the use of axenic culture of living *E. coli* bacteria as a food source for *H. contortus* larvae [36]. Despite improved larval development, the use of live bacteria frequently caused loss of experimental replicates due to bacterial overgrowth (Fig. 1c). In contrast, the addition of heat-treated and lyophilized *E. coli* to the basic medium, as described by Coles and colleagues [39], was sufficient to promote *in vitro* development of larvae to L3 stage (Fig. 1d), if problems associated with bacterial or fungal contamination were avoided. In our experiments, this issue was alleviated by improved egg purification using Percoll gradient and subsequent extensive washes of eggs in antibiotic-antimycotic solution before seeding them in the test plate. This procedure resulted in larval development to L3 of ~75 % in average (Additional file 1: Figure S1) and supported one week cultivation of hatched larvae without inducing bacterial or fungal contamination (Fig. 1d).

Nematotoxic properties of the fungal fruiting body lectins used in our study were previously investigated in the free-living model nematode *C. elegans*. Five of the tested lectins, AAL, CCL2, MOA, CGL2, and Tectonin, demonstrated toxicity against larval stages of the nematode manifesting in inhibition of larval development, which in some cases (MOA, CCL2 and CGL2) resulted in worm death [19, 22–25, 30, 40]. CGL3 lectin had no effect on nematode, insect and protozoan model organisms [24]. Conservation of the target glycan epitopes between *C. elegans* and the parasitic nematodes [21, 27, 40–42] and their location on the intestinal epithelium in *C. elegans*, led us to the hypothesis that the same lectins which are toxic to *C. elegans* might exhibit toxicity to *H. contortus* larval stages and therefore might serve as leads in discovery of novel hidden vaccine candidates. Actually, four of the five lectins toxic to *C. elegans* larvae, AAL, CCL2, MOA, and CGL2 were able to inhibit the development of *H. contortus* larvae *in vitro* in a dose-dependent manner (Fig. 2). However, during one week cultivation only the larvae exposed to MOA lectin died as a result of the treatment, while the other three lectins AAL, CCL2 and CGL2 had a larvistatic effect that manifested as L1 stage arrest (Additional file 2: Figure S2). The results of the lectin toxicity assay on *H. contortus* larvae were in accordance with the data obtained by immunohistochemistry using biotinylated lectins. The gastrodermal tissue of both, *in situ*-labelled larvae and fixed adult sections, stained positive with lectins exhibiting toxicity, while the two lectins which had no effect on the development of larvae, Tectonin and CGL3, were negative (Fig. 2, CGL3 not shown). CGL3 lectin was shown to specifically bind to LacdiNAc (GalNAc-beta1,4-GlcNAc) which was found on N-glycan antenna of insects [43] and recently also in *H. contortus* [26], but exposure of larvae to this lectin was interestingly not toxic. This epitope, in fucosylated and non-fucosylated form, is discussed as a vaccine candidate against schistosomes because it was shown to contribute to the ability of rhesus monkeys to clear the infection [44]. Intriguingly Tectonin, which was toxic to *C. elegans* larvae, had no effect on development of *H. contortus* larvae. Tectonin target are O-methylated mannose and fucose residues on N-glycan antenna in *C. elegans* and it was shown that a functional *samt*-*1* gene, possibly coding for a Golgi SAM-transporter, is required for their biosynthesis [25]. Although a *C. elegans samt*-*1* homolog is present in the genome of *H. contortus* (*P* = 2.6e–164) to date there have been no reports of O-methylated glycans in this parasitic nematode. It is possible that in *H. contortus*, the glycans targeted by CGL3 and Tectonin have a temporal (during larval development) or spatial distribution that prevents them to be targeted by orally ingested lectins. An example of different spatial distribution of conserved glycans between free-living and parasitic nematodes is found in *Toxocara canis* [45] where methylated glycans constitute a part of excretory/secretory antigen, while in *C. elegans* they localize on the gut epithelium and body surface [25]. Based on phenotypic assessment of larval stages and localization of lectin binding sites, we can assume that there is a difference in the mode of toxicity between the lectins. CGL2, CCL2 and AAL recognised epitopes on the apical surface of the gut microvilli of the adults and exposure of larvae to those lectins manifested in growth arrest without killing the larvae during one week of cultivation. The observed phenotype closely resembles the effect of cultivating the larvae in basal media containing no bacteria as food source (Fig. 1a). It is important to note that the function of pharynx seems to be unaffected, as judged by the ability of biotinylated lectins to bind the gut after pharyngeal bulb. Therefore, it is possible that binding of CGL2, CCL2 and AAL lectins to the luminal surface of the gut microvilli interferes with resorption of nutrients in larval stages. These results are in accordance with our recent results on the nematotoxicity of CCL2 in *C. elegans* [30]. In contrast, the larvae exposed to MOA lectin displayed characteristic deformations in morphology during development, manifesting in shrunk body and visibly enlarged intestine after pharynx/bulbus (Additional file 2: Figure S2) and died before reaching the L3 stage. Observed structural deformations of the gut closely resemble those previously described in a *C. elegans* study which demonstrated that MOA-mediated nematotoxicity is directly linked to binding of the lectin to glycosphingolipids of the worm and further biochemical characterization revealed a critical role of cysteine protease activity in this process [19]. The vaccine potential of the glycoepitope targeted by MOA lectin is further confirmed by a study that examined the sera of protected lambs using glycan microarray analysis in which two glycan antigens that possibly contribute to protection against *H. contortus* challenge infection were identified [18]. One of those, the Galα1-3GalNac, is the glycan targeted by MOA lectin. Combining toxicity of MOA to larval stages, protective antibodies in the lamb serum against MOA target glycan and immunohistochemistry studies confirming gut localisation of this epitope in adult worms (and therefore accessibility of the epitope to the specific protective antibodies) it is realistic to conclude that the Galα1-3GalNac epitope presents a promising vaccine candidate against *H. contortus*. Toxicity of CGL2 towards *C. elegans* was shown to be mediated by binding of the lectin to the Gal-β1,4-Fuc-α1,6-epitope (Gal-Fuc epitope) on the proximal GlcNAc residue of N-glycan cores [22]. The Gal-Fuc epitope has been identified in parasitic nematodes *Ascaris suum* and *Oesophagostomum dentatum* [41] and recently, the existence of this and yet another Gal-Fuc epitope have been demonstrated for cores of *H. contortus* N-glycans [26].

Similarly, CCL2 lectin targets α1,3 fucosylated N-glycan cores [23] which have been previously identified as antigens in *H. contortus* [27]. Interestingly, one of the most promising *H. contortus* vaccine candidates, the native aminopeptidase H11 expressed in the worm gut has been shown to contain this glycan modification [29]. Since other expression systems failed to stimulate comparable levels of protection to the native antigen, attempts have been made to produce correctly folded and glycosylated H11 by using *C. elegans* as expression system [28]. This approach, however, did not reduce the *H. contortus* worm burden or egg shedding, possibly because the glycan structures made by *C. elegans* were not completely identical to those of *H. contortus*, or there are additional factors involved in the protective effect of the native H11 antigen preparation. In this regard, the conservation of glycoepitopes between different nematode species might offer the possibility to use a non-parasitic, free-living species as a source of the antigen for a first generation of vaccines.

Little is known about the glycan targets of the AAL lectin in *H. contortus* and *C. elegans*. Genetic data suggests that the core fucose-residues on *C. elegans* N-glycan are not responsible for the nematotoxicity of this fucose-binding lectin [24]. Alternative AAL targets are fucosylated O-glycans which have recently been detected in *C. elegans* [46]. Nothing is known about the structure of O-glycans in *H. contortus* to date.

## Conclusions

In addition to what we know from studies in free-living nematodes, the *in vitro* studies reported here provide novel data regarding the effects of nematotoxic fungal lectins against *H. contortus* larval stages. We show that effector lectins of a fungal defence system targeting conserved glycans in predatory nematodes in nature, may serve as a tool for the identification of potential targets of the vertebrate immune system in parasitic nematodes. The spatial and developmental distribution of the target glycans of the fungal lectins in *H. contortus* reveals them as hypothetical hidden antigens and thus further supports this idea. The question whether these epitopes can be used as effective vaccines against parasitic nematodes, however, is still open and has to be experimentally validated.

## Additional files

Additional file 1: Figure S1. Standardization of the larval development assay. In eighteen independent experiments performed in triplicates under conditions of egg preparation and *in vitro* cultivation of larvae as described in the material and methods section, in average ~75 % of larvae developed to L3 stages during one week cultivation (range 57.57 % – 88.75 %). (TIFF 2385 kb) Additional file 2: Figure S2. Phenotypic changes in *H. contortus* larvae exposed to toxic lectins *in vitro*. Comparison of phenotypic manifestations in *H. contortus* larvae exposed to lectins during one week *in vitro* cultivation. In the low concentration of MOA lectin (1 μg/ml) about 50 % larvae develop to L3 stage, while the affected larvae are shorter and display irregular body morphology. Exposure to higher concentrations of MOA (5 μg/ml) results in death of most of the larvae (≥98 %, note shrunk cuticles and irregular body shapes). Larvistatic effect can be observed in larvae exposed to CCL2, CGL2 and AAL lectins. The larvae survive, but fail to develop to L3 stage (note the difference in size of one larva representing L3 stage compared to the other larvae which are arrested in L1 stage in AAL sample). All images were acquired using the same optical magnification (10x) and size differences are to the scale. Scale bars = 200 μm. (TIFF 2309 kb)
