# Supplementary figures and images for: Inhibition of Haemonchus contortus larval development by fungal lectins
Source: Parasit Vectors. 2015 Aug 19;8:425. doi: 10.1186/s13071-015-1032-x (PMC4539729; doi:10.1186/s13071-015-1032-x)

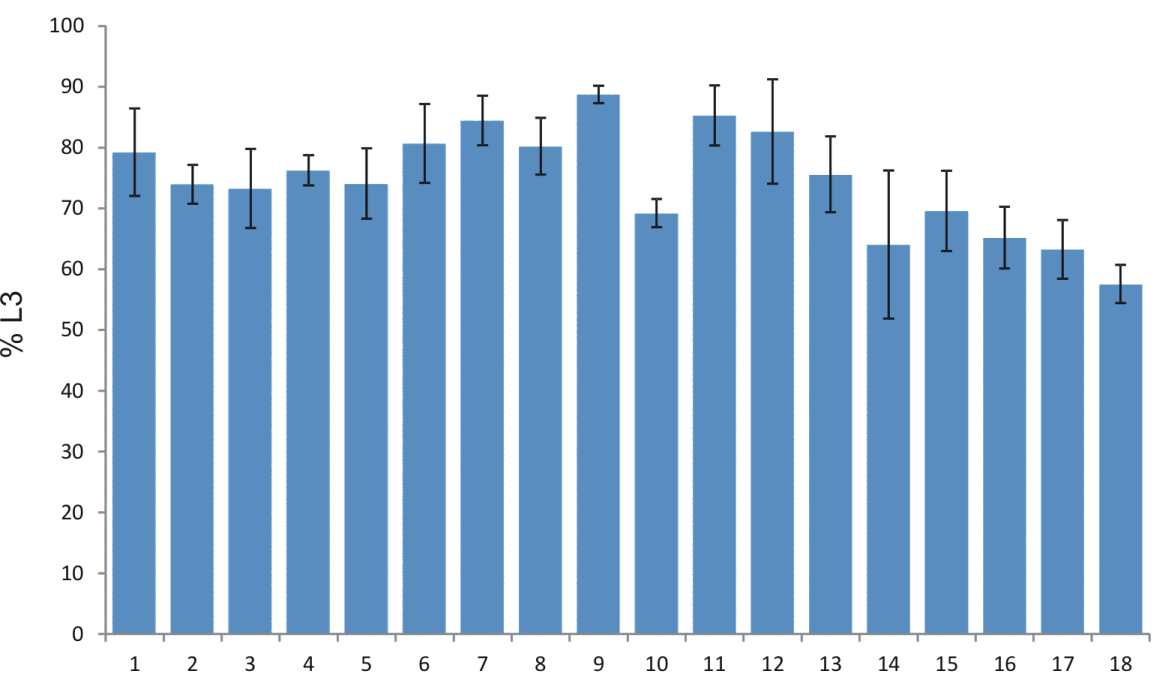

Supplement: Additional file 1: Figure S1. — Standardization of the larval development assay. In eighteen independent experiments performed in triplicates under conditions of egg preparation and in vitro cultivation of larvae as described in the material and methods section, in average ~75 % of larvae developed to L3 stages during one week cultivation (range 57.57 % – 88.75 %). (TIFF 2385 kb) [file 13071_2015_1032_MOESM1_ESM.tif]

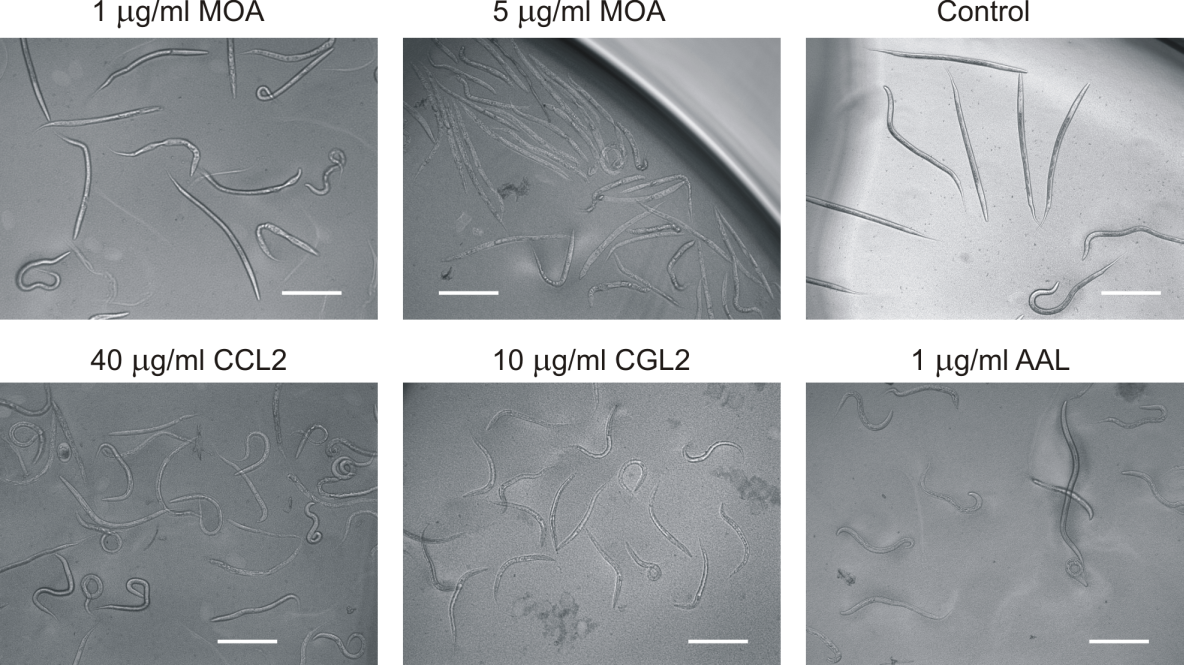

Supplement: Additional file 2: Figure S2. — Phenotypic changes in H. contortus larvae exposed to toxic lectins in vitro. Comparison of phenotypic manifestations in H. contortus larvae exposed to lectins during one week in vitro cultivation. In the low concentration of MOA lectin (1 μg/ml) about 50 % larvae develop to L3 stage, while the affected larvae are shorter and display irregular body morphology. Exposure to higher concentrations of MOA (5 μg/ml) results in death of most of the larvae (≥98 %, note shrunk cuticles and irregular body shapes). Larvistatic effect can be observed in larvae exposed to CCL2, CGL2 and AAL lectins. The larvae survive, but fail to develop to L3 stage (note the difference in size of one larva representing L3 stage compared to the other larvae which are arrested in L1 stage in AAL sample). All images were acquired using the same optical magnification (10x) and size differences are to the scale. Scale bars = 200 μm. (TIFF 2309 kb) [file 13071_2015_1032_MOESM2_ESM.tif]
